# The relationship between G1 (c.260 G>A) and G4 (c.721 G>A) polymorphisms in the GDF9 gene and the litter size of sheep: A meta-analysis study

**DOI:** 10.5455/javar.2023.j715

**Published:** 2023-12-31

**Authors:** Agung Budiyanto, Slamet Hartanto, Rini Widayanti, Erif Maha Nugraha Setyawan, Heru Ponco Wardono, Sri Gustari

**Affiliations:** 1 Department of Reproduction, Obstetrics and Gynecology, Faculty of Veterinary Medicine, Gadjah Mada University, Yogyakarta, Indonesia; 2 National Research and Innovation Agency (BRIN), Jakarta, Indonesia; 3 Department of Biochemistry and Molecular Biology, Faculty of Veterinary Medicine, Universitas Gadjah Mada, Yogyakarta, Indonesia

**Keywords:** Fecundity Gene, Litter Size, Ovis aries, Single Nucleotide Polymorphism

## Abstract

**Objective:**

The results of G1 and G4 polymorphisms as litter-size (LS) markers of ewes remain contradictory. The aim was to evaluate the impact of G1 (c.260 G>A) and G4 (c.721 G>A) polymorphisms on the LS of sheep by synthesizing data from multiple previous studies.

**Methods:**

Data were extracted from 14 eligible articles. The genotypes of G1 and G4 polymorphisms were homozygous wild-type (WW), heterozygous (WM), and homozygous mutant-type (MM). The standardized mean difference (SMD) method using random effect models was employed to determine the effect size of G1 and G4 polymorphisms on LS under dominant, recessive, additive, and co-dominant genetic models. Heterogeneity was analyzed with the I2 statistic index. Publication bias was depicted with funnel plots and tested by Egger’s and Begg’s tests.

**Results:**

The study showed that the correlation between G1 polymorphism and LS in sheep was not significant (p > 0.05) under all genetic models. The influence of G4 polymorphism on the LS of sheep was found significantly (p < 0.05) under dominant [SMD = 0.28, I2 = 0% (no heterogeneity)] and co-dominant [SMD = −0.14, I2 = 36% (moderate heterogeneity)] genetic models. The WM genotype of G4 polymorphism increased LS, while the MM genotype reduced LS in sheep. Publication bias among G1 and G4 polymorphism studies was absent in all genetic models.

**Conclusion:**

Thus, the study revealed that G4 polymorphism could be a potential genetic marker for LS in ewes. On the contrary, G1 polymorphism has no association with the LS of ewes.

## Introduction

Domesticated sheep are small ruminants with essential values in society’s lives [1], such as providing food and wool, economic sources, cultural and religious festivities, and other roles [2,3]. Reproductive traits play a critical role in the sheep industry [4]. They have an impact on sustainability and profitability [5,6]. Litter size (LS), the number of offspring born to a female animal at a single birth, is one of the crucial reproductive traits of livestock production, especially in the sheep industry [7]. It affects the profitability and efficiency of sheep production [8]. The increment in LS from 1.0 to 2.2 lambs per ewe is linear with the increase in gross margin revenue in the sheep industry [9]. Genetic factors are fundamental for LS, besides nutrition and environmental conditions. Several fecundity genes have been identified as valuable genetic markers because of their effect on the LS of ewes [10]. Exploring specific genes as LS markers is necessary to enhance sheep’s productivity.

Growth differentiation factor 9 (*GDF9*) is renowned for crucially regulating ovarian follicle development in mammals, including the growth of oocytes and granulosa cells [11] and theca cell proliferation [12]. Oocytes primarily secrete *GDF9* [13]*. GDF9* has crucial functions in ovulation and fertilization [14]. In sheep, the *GDF9* gene was found on chromosome 5 [15], which comprises two exons and one intron. The *GDF9* gene has been identified as the putative marker for the prolificacy of sheep and goats [16–19]. *G1* (*c.260 G>A*) and *G4* (*c.721 G>A*) polymorphisms in the *GDF9* gene are the most studied loci in the *GDF9* gene used to investigate the fertility traits of sheep based on the prolificacy parameter. *G1* causes the alteration of arginine for histidine (*R87H*) at coding residue 87 in exon 1 and *G4* changes glutamic acid to lysine (*E241K*) at coding residue 241 [20,21]. However, the results of *G1* and *G4* polymorphisms on the LS of ewes remain contradictory. Several studies reported that *G1* and *G4* polymorphisms have affected the LS of sheep [22–29]. Meanwhile, there are other investigations that discovered no association between *G1* and *G4* polymorphisms and LS of sheep [30–34]. Therefore, advanced statistical analysis is required to elucidate the impact of *G1* and *G4* polymorphisms on the LS of ewes based on the findings of previous studies.

Meta-analysis is a systematic study designed to statistically synthesize the data of multiple previous studies to derive a quantified conclusion [35]. It enables researchers to integrate the data from insufficiently powered experiments to generate conclusive findings [36]. Because of a data-pooled study, the result of a meta-analysis study may be more precise and robust than the individual study [37]. Numerous meta-analysis studies have been employed to assess the influence of the fecundity genes *BMP15* and *BMPR1B* polymorphisms on the LS of sheep and goats [38,39] and the relationship between *c.1189 G>A* variation in the *GDF9* gene and the LS of dose [40]. To the best of our investigation, no meta-analysis examining the correlation between the *G1* and *G4* polymorphisms in the *GDF9* gene and the LS of sheep has been found. Thus, the aim was to evaluate the link between *G1* and *G4* polymorphisms in the *GDF9* gene and the LS of sheep by synthesizing data from numerous published articles.

## Materials and Methods

### Literature search strategy

The meta-analysis study used preferred reporting items for systematic reviews and meta-analyses (PRISMA) guidelines to qualify the eligible studies [41]. Multiple academic databases, including Google Scholar, Science Direct, Wiley, and Springer, were independently employed to retrieve the related studies by two scientists (AB and SH) with several keywords, alone or in combination, as follows: “*GDF9,*” “Polymorphism,” “SNP,” “Prolificacy,” “Fertility,” “LS,” and “Sheep.” Moreover, we verified that no articles were missed by scrutinizing the reference list of extracted articles. All differences concerning the eligibility of the studies were settled through discussions. Finally, if no agreement regarding the inclusion of eligible studies was obtained, a discussion with a third scientist (RW) was used to resolve all remaining differences.

### Inclusion and exclusion criteria

The eligible studies were included if they were: (1) studying *G1* (*c.260 G>A*) and *G4* (*c.721 G>A*) polymorphisms in sheep; (2) each genotype is provided with the number of samples; (3) each genotype displays the least-squares mean (LSM); (4) evaluating the correlation *G1* and *G4* polymorphisms and LS; and (5) reporting a standard error (SE) or standard deviation (SD) or confidence interval (CI). The following criteria apply to excluded studies: (1) non-English-language articles; (2) no full text is available; (3) relevant data are insufficient; (4) duplicated studies; and (4) review studies.

### Data extraction

Selected studies were extracted and input as follows: the first author’s name, publication’s year, location of the study, sheep breed, the number of samples, LSM, SD, and significant level. An online extractor, WebPlotDigitizer (https://apps.automeris.io/wpd/), was applied to extract the LSM and SE from the graphed data [42]. To obtain SD from sample sizes and SE of LSM from each genotype, the following formula was employed: SD = SE, where *N* is the number of samples from each genotype. Meanwhile, SD from the number of samples and 95% CI from each genotype were computed with the following formula: SD = *x* (upper limit—lower limit)/3.92 [43].

**Figure 1.**
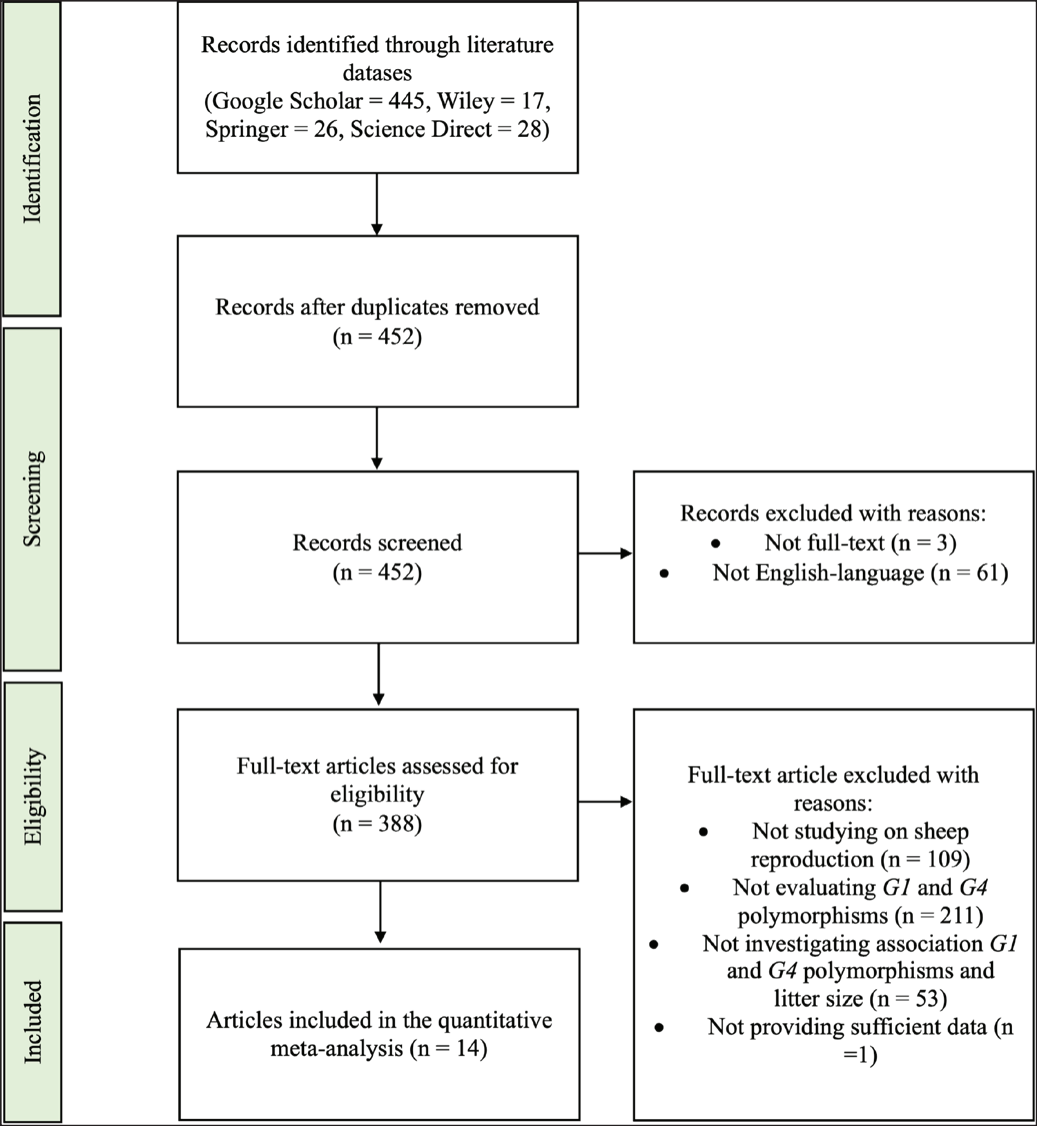
The flow chart based on PRISMA guidelines illustrates the comprehensive process for screening included and excluded studies.

### Statistical analysis

The study used Review Manager v5.4 software and the “meta” package of R v4.2.2 software [44]. Data were synthesized with the standardized mean difference (SMD) method [41] using random-effects models under dominant (*WW* + *WM vs. MM*), recessive (*WW vs. WM + MM*), additive (*WW vs. MM*), and co-dominant (*WW + MM vs. WM*) genetic models to determine the effect size of *G1* and *G4* polymorphisms on the LS of ewes. The *p*-values <0.05 indicated the effect was considered to be significant.

To test heterogeneity among studies, the *I^2^* statistic index was applied. The *I^2^* values <25%, *I^2^* values between 25% and 50%, and *I^2^* values >50% indicate low, moderate, and high heterogeneity, respectively [45]. Moreover, we performed a sensitivity test to investigate the firmness of all results by erasing a single study of each genetic model at a time.

Publication bias among the studies was depicted using funnel plots and tested using Egger’s and Begg’s tests. The presence of an asymmetric funnel plot and the *p*-value ≤0.05 of Egger’s and Begg’s demonstrate the high risk of publication bias [46].

## Results

### Characteristics of included eligible studies

The present study applied a PRISMA flow diagram to display the comprehensive process of selecting qualified research articles for a meta-analysis, as illustrated in Figure 1. A comprehensive search of multiple literature databases identified a total of 516 articles. After eliminating 64 duplicate papers, the remaining 452 publications were screened to eliminate those that did not provide full-text articles and no English-language articles, resulting in 388 articles for the eligibility assessment.

After applying the exclusion criteria, 373 articles were removed. One study was rejected because it did not provide SE, SD, or CI. Finally, 14 eligible articles were selected for the meta-analysis study. Table 1 displays the characteristics of eligible studies for our meta-analysis study.

**Table 1.** The characteristics of included studies in this meta-analysis study.

Study	Year	Country	Breed	Total sample	Genotypes^a^			LSM ± SD^b^			Sig.
					*WW*	*WM*	*MM*	*WW*	*WM*	*MM*	
*G1* (*c.260 G>A*) polymorphism											
Abdelgadir et al. [22]	2021	Sudan	Sudanese Desert	88	52	30	6	1.248 ± 0.238	1.386 ± 0.252	1.040 ± 0.230	yes
Aboelhassan et al. [23]	2021	Egypt	Egyptian	95	92	3	*NE*	2.170 ± 4.220	0.048 ± 0.064	*NE*	yes
Hossain et al. [50]	2020	Bangladesh	Bangladesh	126	65	57	4	1.590 ± 0.726	1.830 ± 0.755	2.000 ± 0.820	yes
Rezaei et al. [26]	2020	Iran	Iran-Black	114	63	45	6	1.650 ± 0.714	1.890 ± 0.604	1.880 ± 0.392	yes
Pineda et al. [33]	2018	Colombia	Colombia Hair	150	123	27	*NE*	1.240 ± 0.300	1.330 ± 0.360	*NE*	no
Talebi et al. [34]	2018	Iran	Mehraban	115	82	31	2	1.160 ± 0.873	1.170 ± 0.856	1.000 ± 0.252	no
Eghbalsaied et al. [31]	2017	Iran	Afshari	145	118	20	7	1.082 ± 0.554	1.326 ± 0.936	1.005 ± 0.027	no
Eghbalsaied et al. [31]	2017	Iran	Ghezel	126	93	30	3	1.611 ± 0.779	1.498 ± 0.767	1.999 ± 0.019	no
Eghbalsaied et al. [31]	2017	Iran	Lori-Bakhtiyari	171	153	18	*NE*	1.578 ± 0.819	1.772 ± 0.609	*NE*	no
Eghbalsaied et al. [31]	2017	Iran	Shal	54	44	5	5	1.549 ± 0.793	1.331 ± 0.755	1.668 ± 0.732	no
Gorlov et al. [24]	2018	Russia	Salsk	500	440	60	*NE*	1.130 ± 1.888	1.800 ± 0.929	*NE*	yes
Gorlov et al. [24]	2018	Russia	Volgograd	500	420	80	*NE*	1.220 ± 2.254	1.180 ± 1.521	*NE*	yes
Jawasreh et al. [28]	2017	Jordan	Romanov	70	41	29	*NE*	2.732 ± 1.088	1.940 ± 0.969	*NE*	yes
Abdoli et al. [30]	2013	Iran	Mehraban	85	24	53	8	1.245 ± 0.315	1.103 ± 0.339	1.047 ± 0.205	no
Liandris et al. [32]	2012	Greece	Chios	239	156	62	21	1.590 ± 2.403	1.450 ± 1.615	2.250 ± 1.076	yes
Liandris et al. [32]	2012	Greece	Karagouniki	259	250	9	*NE*	1.320 ± 1.766	1.070 ± 0.507	*NE*	no
Moradband et al. [25]	2011	Iran	Baluchi	134	96	27	11	1.238 ± 0.314	1.386 ± 0.265	1.032 ± 0.312	yes
Roy et al. [27]	2011	India	Bonpala	97	86	10	1	1.670 ± 0.620	1.900 ± 0.570	1.000 ± 0.000	yes
Javanmard et al. [29]	2011	Iran	Fat-tailed	96	58	38	*NE*	1.160 ± 0.381	1.780 ± 0.432	*NE*	yes
*G4* (*c.721 G>A*) polymorphism											
Aboelhassan et al. [23]	2021	Egypt	Egyptian	95	21	74	*NE*	0.300 ± 0.825	1.950 ± 4.731	*NE*	yes
Eghbalsaied et al. [31]	2017	Iran	Afshari	145	136	9	*NE*	1.240 ± 0.854	1.004 ± 0.031	*NE*	no
Eghbalsaied et al. [31]	2017	Iran	Ghezel	126	79	38	9	1.559 ± 0.858	1.665 ± 0.892	1.665 ± 0.985	no
Eghbalsaied et al. [31]	2017	Iran	Lori-Bakhtiyari	171	105	49	17	1.560 ± 0.768	1.520 ± 0.630	1.330 ± 0.660	no
Eghbalsaied et al. [31]	2017	Iran	Shal	54	28	24	2	1.753 ± 0.668	1.389 ± 0.569	1.500 ± 0.693	no
Gorlov et al. [24]	2018	Russia	Salsk	500	*NE*	60	440	*NE*	1.800 ± 0.930	1.130 ± 1.888	yes
Gorlov et al. [24]	2018	Russia	Volgograd	500	*NE*	80	420	*NE*	1.880 ± 1.521	1.220 ± 2.254	yes
Liandris et al. [32]	2012	Greece	Chios	239	154	79	6	1.830 ± 2.136	2.170 ± 1.518	1.300 ± 0.705	no
Liandris et al. [32]	2012	Greece	Karagouniki	259	197	49	13	1.130 ± 1.779	1.200 ± 0.975	1.260 ± 0.629	no
Talebi et al. [34]	2018	Iran	Mehraban	115	78	33	4	1.180 ± 0.601	1.160 ± 0.582	1.000 ± 0.236	no
Roy et al. [27]	2011	India	Bonpala	97	86	10	1	1.670 ± 0.620	1.900 ± 0.570	1.000 ± 0.000	no

^a^*WW*, homozygous wild-type genotype; *WM*, heterozygous genotype; *MM*, homozygous mutant-type genotype, ^b^LSM, least-squares mean; SD, standard deviation; NE, no existence; Sig., significance.

**Table 2.** Summary of results of the meta-analysis on the relationship between *G1* (*c. 260 G>A*) and *G4* (*c.721 G>A*) polymorphisms in the *GDF9* gene and LS of sheep.

SNP^a^	Genetic model^b^	Number of cohort	Test of relationship^c^		*p*-value	Test of heterogeneity^d^		
			SMD	95% CI		Model	*p*-value	*I* ^2^
*G1* (*c.260 G>A*)	Dominant (*WW* + *WM vs. MM*)	11	0.13	−0.20; 0.47	ns	R	0.08	41%
	Recessive (*WW vs. WM* + *MM*)	19	−0.13	−0.33; 0.08	ns	R	<0.0001	74%
	Additive (*WW vs. MM*)	11	0.12	−0.21; 0.44	ns	R	0.12	35%
	Co-dominant (*WW + MM vs. WM*)	19	−0.01	−0.25; 0.22	ns	R	<0.0001	79%
*G4* (*c.721 G>A*)	Dominant (*WW* + *WM vs. MM*)	9	0.28	0.13; 0.43	<0.01	R	0.87	0%
	Recessive (*WW vs. WM* + *MM*)	9	−0.03	−0.15; 0.10	ns	R	0.26	20%
	Additive (*WW vs. MM*)	7	0.14	−0.14; 0.43	ns	R	0.86	0%
	Co-dominant (*WW + MM vs. WM*)	11	−0.14	−0.27; −0.01	0.04	R	0.11	36%

^a^SNP, single nucleotide polymorphism, ^b^*WW*, homozygous wild-type genotype; *WM*, heterozygous genotype; *MM*, homozygous mutant-type genotype, ^c^SMD, standardized mean difference; CI, confidence interval. ^d^R, random-effect models.

### Correlation between the G1 and G4 polymorphisms and LS of sheep

**Figure 2.**
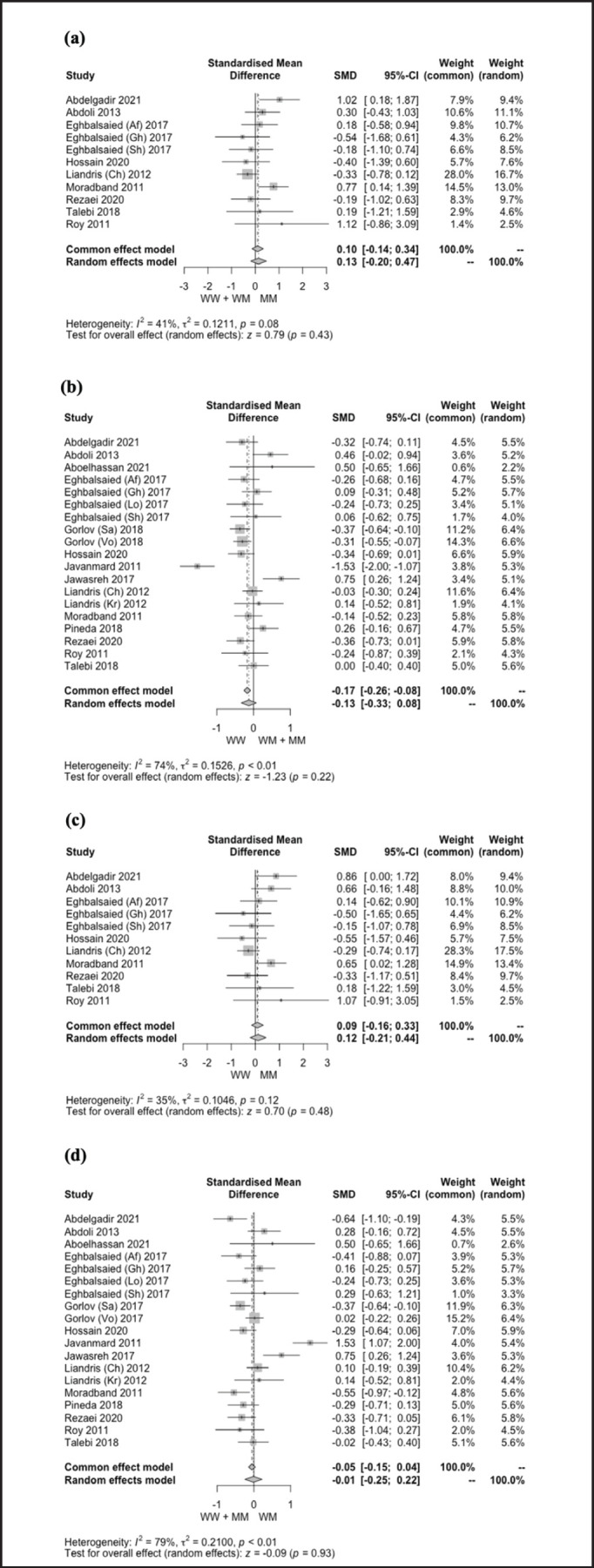
Forest plot showing the association between *G1* (*c.260 G>A*) polymorphism in the *GDF9* gene and LS of sheep under dominant (a), recessive (b), additive (c), and co-dominant (d) genetic models.

**Figure 3.**
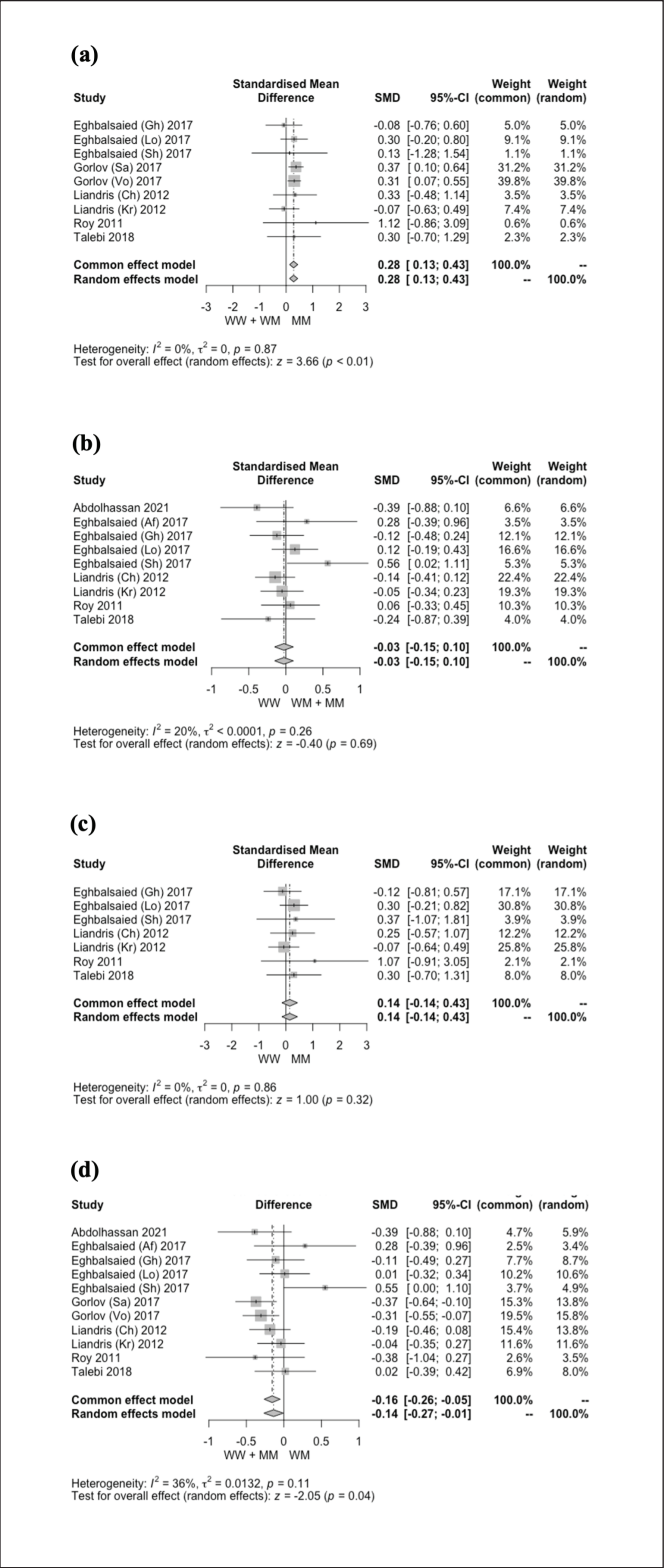
Forest plot displaying the relationship between *G4* (*c.721 G>A*) polymorphism in the *GDF9* gene and LS of sheep under dominant (a), recessive (b), additive (c), and co-dominant (d) genetic models.

A comprehensive summary of the meta-analysis outcomes about the relationship between *G1* and *G4* polymorphisms in the *GDF9* gene and the LS of sheep is shown in Table 2. Figures 2 and 3 display the forest plots, illustrating meta-analysis findings on the effect of *G1* and *G4* polymorphisms on the LS of sheep. The result revealed that *G1* polymorphism had no significant effect (*p* > 0.05) on the LS of sheep under all genetic models. On the contrary, the LS of sheep was significantly affected (*p* < 0.05) by *G4* polymorphism under dominant (SMD = 0.28) and co-dominant (SMD = −0.14) genetic models. In addition, the effect of *G4* polymorphism on LS was not observed (*p* > 0.05) under recessive (SMD = −0.03) and additive (SMD = 0.14) genetic models.

### Heterogeneity, sensitivity, and publication bias analysis

**Figure 4.**
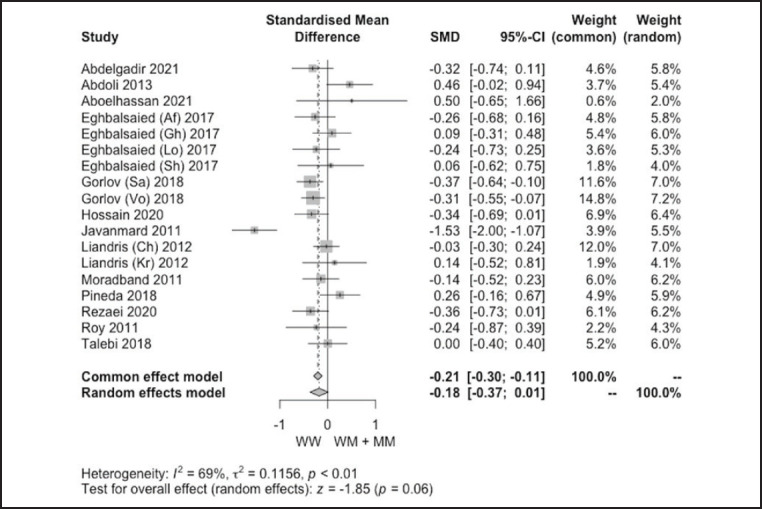
Forest plot showcasing sensitivity analysis of the effect of *G1* (*c.260 G>A*) polymorphism on LS of sheep under the recessive genetic model after removing Jawasreh et al. [28] studies (compared with Fig. 2b).

Moderate (25% < *I^2^* ≤ 50%) to high (*I^2^* > 50%) heterogeneity was found among *G1* polymorphism studies. The *I^2^*-value under dominant, recessive, additive, and co-dominant genetics models among *G4* polymorphism studies was 0% (no heterogeneity), 20.1% (low heterogeneity), 0% (no heterogeneity), and 36% (moderate heterogeneity), respectively. Systematically erasing one study at a time and applying it to all models was the sensitivity test used to clarify the robustness of the pooled effect size. A significant change was found under the dominant genetic model in the *G1* study after removing Jawasreh et al.’s [28] reported *G1* polymorphism in Egyptian sheep, as shown in Figure 4. No significant change under all genetic models was found in the *G4* polymorphism study.

**Figure 5.**
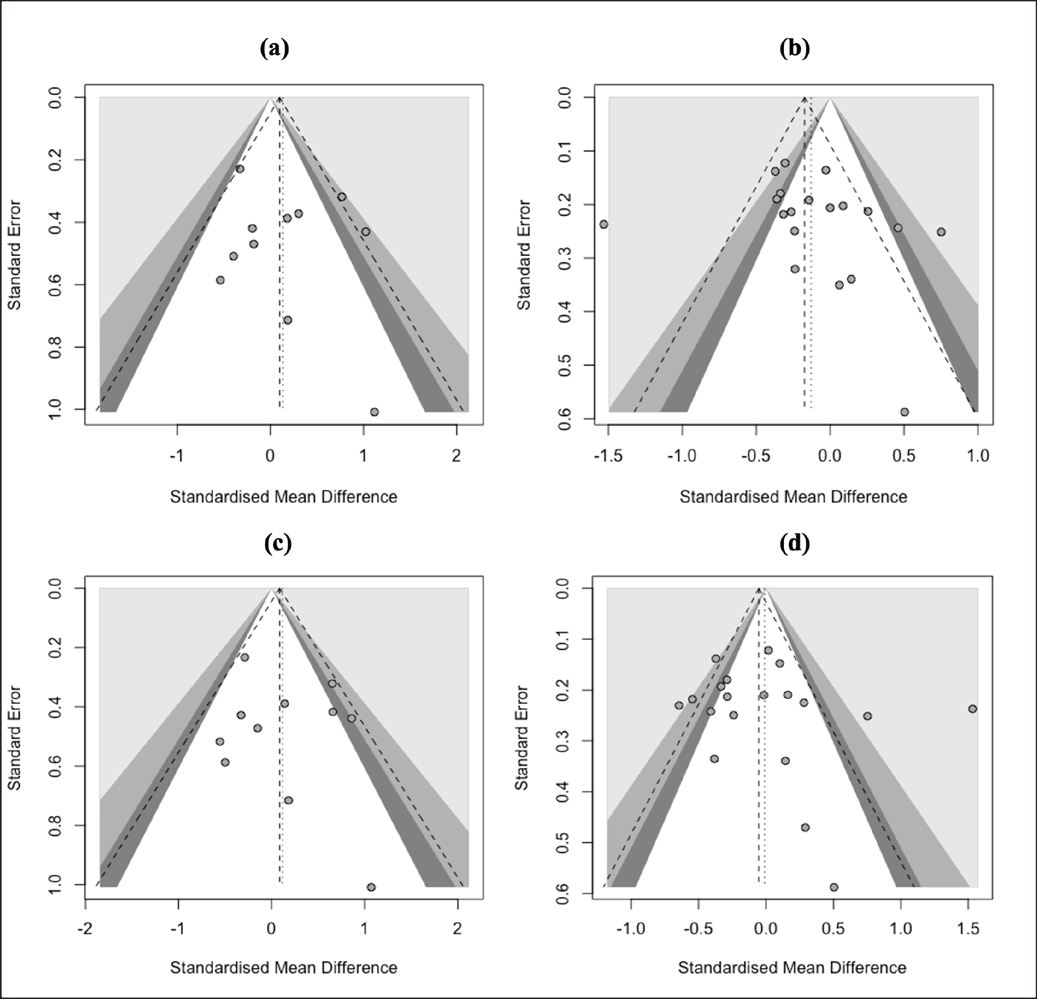
Funnel plots exhibiting analysis of publication bias among *G1* (*c.260 G>A*) polymorphism studies under the dominant model (a), recessive model (b), additive model (c), and co-dominant model (d).

**Figure 6.**
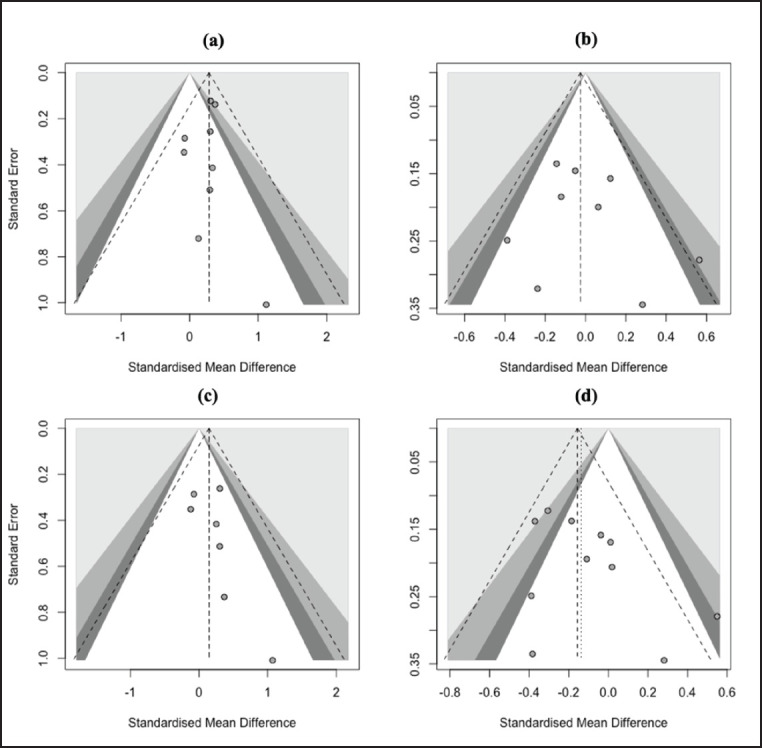
Funnel plots depicting analysis of publication bias among *G4* (*c.721 G>A*) polymorphism studies under the dominant model (a), recessive model (b), additive model (c), and co-dominant model (d).

Figures 5 and 6 display the results of the publication bias test, as depicted by the funnel plots. No asymmetrical plots in all genetic models indicated publication bias was absent in the *G1* and *G4* studies. Egger’s and Begg’s tests were employed for all genetic models to confirm the funnel plot results. A significant publication bias in all models was also not found (*p* > 0.05) in both studies, as shown in Table 3.

## Discussion

The *GDF9* gene has been identified as a highly prolific gene in sheep, along with *BMP15* and *BMPR1B* [10]. *GDF9* gene single nucleotide polymorphisms (SNPs) affect the fertility traits of sheep [47]. The *G1* polymorphism (*c.260 G>A*) and *G4* (*c.721 G>A*) polymorphisms in the *GDF9* gene have been intensively studied to characterize the LS of sheep. However, the reported findings are still conflicting. Abdelgadir et al. [22] demonstrated that the *G1* polymorphism effect significantly affected the LS of the Sudanese desert sheep. Moreover, Aboelhassan et al. [23] and Gorlov et al. [24] demonstrated that the correlation between *G1* and *G4* mutations and LS in ewes was significant. However, Abdoli et al. [30], Eghbalsaied et al. [31], and Talebi et al. [34] revealed that the *G1* and *G4* polymorphisms had no association with the LS of sheep.

**Table 3.** Summary of the risk of publication bias detected by Egger’s and Begg’s tests.

SNP^a^	Genetic model^b^	*I* ^2^	*p*-value^c^	
			Egger’s test	Begg’s test
*G1* (*c.260 G>A*)	Dominant (*WW* + *WM vs. MM*)	41%	0.56	0.69
	Recessive (*WW vs. WM* + *MM*)	74%	0.33	0.06
	Additive (*WW vs. MM*)	35%	0.58	0.82
	Co-dominant (*WW + MM vs. WM*)	79%	0.48	0.31
*G4* (*c.721 G>A*)	Dominant (*WW* + *WM vs. MM*)	0%	0.62	0.83
	Recessive (*WW vs. WM* + *MM*)	20%	0.52	0.53
	Additive (*WW vs. MM*)	0%	0.22	0.18
	Co-dominant (*WW + MM vs. WM*)	36%	0.12	0.12

^a^SNP, single nucleotide polymorphism, ^b^WW, homozygous wild-type genotype; *WM*, heterozygous genotype; *MM*, homozygous mutant-type genotype. ^c^*p*-value >0.05, no publication bias; *p*-value <0.05, publication bias high.

To settle the contradictory study results, meta-analysis has the ability to improve the precision and accuracy of scientific results by integrating and synthesizing pooled data from multiple studies. It provides robust and reliable conclusions that have positive impacts on many scientific studies [48]. Numerous studies have employed meta-analysis to evaluate the impact of fecundity gene polymorphisms on the LS of sheep and goats [39–41]. However, to the best of our knowledge, this study was the first comprehensive meta-analysis to investigate the association between *G1* and *G4* polymorphisms and the LS of sheep. We synthesized data from 14 articles under dominant, co-dominant, recessive, and additive genetic models.

This study demonstrated that the *G1* polymorphism had no significant effect on the LS of sheep. However, the *G4* mutation affected the LS, whereby the homozygous mutant-type (*MM*) genotype reduced the LS and the heterozygous (*WM*) genotype increased the LS of ewes. It is assumed that the heterozygous genotype of the G4 polymorphism in the GDF9 gene results in high ovulation rates, leading to higher LS. Hanrahan et al. [21] reported that the heterozygous genotype of the GDF9 gene boosts the ovulation rate. This finding was similar to the meta-analysis study conducted by Mahmoudi et al. [40] reported that *c. 1118 G>A* polymorphism of the *GDF9* gene had significantly affected the LS of goats, whereby the heterozygous genotype had a positive effect on LS.

Furthermore, no publication bias and moderate heterogeneity under the co-dominant genetic model assure an accurate estimate of the effect size in this study. No publication bias and low heterogeneity confirmed reliable effect sizes in the meta-analysis study [49].

However, it is assumably necessary to re-confirm our findings in future research because the present study has limitations. The several limitations of this study are as follows: a) the small number of included studies; b) the sample size was limited; and c) there was no sub-analysis based on the region of the sheep breed. The future study will focus on adding larger sample sizes to address the limitations of this study.

## Conclusion

Thus, the study revealed that *G4* polymorphism could be a potential genetic marker for LS in ewes. On the contrary, *G1* polymorphism has no association with the LS of ewes. However, the limitations of this study should be considered for further studies.

## List of abbreviations

*BMP15*, bone morphogenetic protein 15; *BMPR1B*, bone morphogenetic protein receptor type-1B; CI, confidence interval; *G1*, c.260 G>A mutation point in *GDF9* gene; *G4*, c.721 G>A mutation point in GDF9 gene; *GDF9*, growth differentiation factor 9; *I**^2^*, I statistic index LS, litter size; LSM, least mean square; *MM*, homozygous mutant-type genotype; PRISMA, Preferred reporting items for systematic reviews and meta-analyses; R, random-effect models; SD, standard deviation; SE, standard error; SMD, standardized mean difference; SNP, single nucleotide polymorphism; *WM*, heterozygous genotype; *WW*, homozygous wild-type genotype.
